# Extending Ripley’s K-Function to Quantify Aggregation in 2-D Grayscale Images

**DOI:** 10.1371/journal.pone.0144404

**Published:** 2015-12-04

**Authors:** Mohamed Amgad, Anri Itoh, Marco Man Kin Tsui

**Affiliations:** 1 Okinawa Institute of Science and Technology (OIST) Graduate University, Okinawa, Japan; 2 Faculty of Medicine, Cairo University, Cairo, Egypt; Deutsches Zentrum für Neurodegenerative Erkrankungen e.V., GERMANY

## Abstract

In this work, we describe the extension of Ripley’s K-function to allow for overlapping events at very high event densities. We show that problematic edge effects introduce significant bias to the function at very high densities and small radii, and propose a simple correction method that successfully restores the function’s centralization. Using simulations of homogeneous Poisson distributions of events, as well as simulations of event clustering under different conditions, we investigate various aspects of the function, including its shape-dependence and correspondence between true cluster radius and radius at which the K-function is maximized. Furthermore, we validate the utility of the function in quantifying clustering in 2-D grayscale images using three modalities: (i) Simulations of particle clustering; (ii) Experimental co-expression of soluble and diffuse protein at varying ratios; (iii) Quantifying chromatin clustering in the nuclei of *wt* and *crwn1 crwn2* mutant *Arabidopsis* plant cells, using a previously-published image dataset. Overall, our work shows that Ripley’s K-function is a valid abstract statistical measure whose utility extends beyond the quantification of clustering of non-overlapping events. Potential benefits of this work include the quantification of protein and chromatin aggregation in fluorescent microscopic images. Furthermore, this function has the potential to become one of various abstract texture descriptors that are utilized in computer-assisted diagnostics in anatomic pathology and diagnostic radiology.

## Introduction

Spatial point analysis, the analysis of dispersion and clustering of events, has been well-studied and heavily utilized in many fields [1,2]. One of the earliest attempts at providing a solid mathematical foundation to quantify “significant” clustering or dispersion was that of Clark and Evans in 1954, which relied on the “average distance to the nearest neighbor” [3]. However, the problem with Clark’s method is that it does not quantify clustering at different scales, unlike other methods that have been later devised to address this issue.

**Fig 1:**
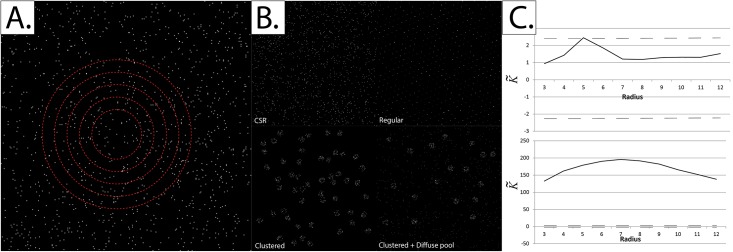
The concept behind Ripley’s K-function. *Panel A*: Ripley’s K-function is defined as the average number of events within a predefined radius of any event, normalized for the event intensity (density) over the whole field of view. A set of radii is typically used to quantify the scale at which clustering (or dispersion) of events occurs (red circles). *Panel B*: Events within a given field of view could be distributed in many forms, including Complete Spatial Randomness (CSR), regularity and clustering (with or without a diffuse pool). *Panel C*: $\tilde{K}$ plots for two of the event distributions in Panel B: CSR (*upper*) and Clustering (*lower*). The dashed lines represent the upper and lower critical quantiles (Q_01_ and Q_99_).

Nowadays, Ripley’s K-function, developed in 1977, is considered to be the “golden standard” in spatial point analysis [4]. Since its original development, Ripley’s K-function has been extensively used in a very large spectrum of applications, including geography, epidemiology, economics and biomedical research [1,2,5–8]. The concept behind Ripley’s K-function is rather simple; it is the average number of events (particles) located within a predefined radius of any typical event, normalized for the event intensity (density) over the same field of view. Ripley’s K-function compares the distribution of events in a given field of view to Complete Spatial Randomness (CSR), otherwise known as a “homogeneous Poisson process”. At CSR, the locations of events are completely random; not regular but random (Fig 1).

Previous attempts to integrate Ripley’s K-function into image processing include its utilization in concrete (segmentation-based) statistics [9,10]. In this paper, we test and optimize the use of Ripley’s K-function as an abstract index of clustering, to allow for event overlap at very high densities. As will be described later, problematic edge effects become of particular relevance at very high densities, and previously-reported patterns linking the radius at which the K-function is maximized with the underlying cluster radius break down. But why would it be useful to extend Ripley’s K-function to allow for event overlap? Our main motivation was to develop an image processing approach to quantifying protein aggregation in fluorescent microscopic images.

While the quantification of higher order protein homo-oligomerization and aggregation propensity *in-vitro* has been thoroughly developed over many decades of protein biochemistry research, the quantification of *in-vivo* oligomerization or aggregation has received relatively less attention. Over the past few years, however, the importance of *in-situ* characterization of protein solubility and aggregation became increasingly clear [11]. This paradigm shift has been caused by a number of developments in protein research including: **a)** a better understanding of the role played by complex intra-cellular environments in altering reaction equilibria and causing a disparity between *in-vitro* and *in-vivo* assays [12,13], **b)** a wealth of evidence outlining the crucial involvement of protein aggregation in a multitude of diseases, including Alzheimer's disease, Huntington's disease, Amyotrophic Lateral Sclerosis (ALS), Parkinson's disease and Prion disease [14], **c)** an increasingly widening scope of protein aggregation research to include emerging fields such as ageing research [15,16], and **d)** the realization that bacterial inclusion bodies (IB's) may be used as sources of active recombinant proteins [17] as well as a model system for amyloid aggregation studies [18].

One of the most problematic issues that arise when trying to quantify *in-vivo* aggregation of proteins is the interference caused by the soluble pool of proteins with accurate and specific identification and characterization of aggregates. Several experimental approaches have been devised to overcome this limitation, and fall into four broad categories, reviewed in a recent commentary by Ami *et al* [11]: Genetically-encoded fusion tags such as GFP or tetra-Cys tags; Conformation-sensitive dyes such as Thioflavin-S; Direct spectroscopic methods such as Nuclear Magnetic Resonance (NMR); Aggregation-sensitive reporters. The majority of these experimental approaches rely on parameters that can differentiate between soluble an aggregated forms of proteins.

Our focus is on the first category: fluorescence microscopy. When fluorescent aggregates are formed as a result of defective protein folding, it has been shown that in some situations it may be possible to quantify aggregation propensity simply by measuring the bulk cell fluorescence of GFP-fusion constructs. That is, since unfolded GFP does not fluoresce while folded GFP (in non-aggregated mutants) emits a detectable fluorescent signal [19]. Other interesting approaches to detect misfolded (or partially folded) aggregates through fluorescence microscopy include the pioneering work of Ignatova *et al* showing that tetra-Cys fusion tags form hyper-fluorescent aggregates, enabling the detection of protein stability and aggregation *in-vivo* in real time [20]; But what about situations in which aggregation and misfolding are not necessarily coupled?

Oftentimes, proteins *in their native state* oligomerize and form cytoplasmic puncta which may or may not be of regular sizes and shapes, and which may constitute only a fraction of the amount of protein in the cell, the rest being in diffuse (soluble) form [21–23]. In such cases, studies of protein supramolecular complexes or aggregates traditionally involved *in-vitro* biochemical methods such as chromatographic and centrifugation assays to separate aggregates from soluble protein pools. The problem, however, is that these biochemical techniques are often prone to *in-vitro* artifacts.

Generally speaking, there are two broad categories of calculations that can be applied to two-dimensional fluorescence microscopic images: "concrete" (segmentation-based) statistics and "abstract" (e.g. texture) statistics [24]. Concrete statistics try to separate out objects from background (segmentation), allowing further calculations to be performed on the objects such as total intensity, volume, localization and so on. Depending on the context, the most suitable segmentation techniques may vary considerably [25]. We faced some difficulty when we tried to quantify the effect of various mutations on the incorporation of a protein, *Cubitus interruptus* (Ci), into cytoplasmic puncta using confocal fluorescence microscopy. The differentiation between punctate and diffuse protein forms in our case was often tricky due to the following factors: **a)** the presence of a soluble pool introduces high background, reducing signal-to-background ratio; **b)** the amount of soluble protein is variant in different regions, causing high variability in background intensity; **c)** clumping of aggregates introduces further ambiguity into the image segmentation process. Besides, the highly heterogeneous nature of the aggregates prevents filtering based on a common morphology, as would have been possible for tube-like [26] or sheet-like [27] structures, for example.

Can an abstract statistical metric be used to quantify protein aggregation? Because of the low signal-to-background ratio and the inherent variability in background in images of protein aggregation studies, isolating aggregates reliably using concrete statistical measures (i.e. segmentation) may sometimes be very difficult. The golden standard, of course, would be to devise better experimental methods that enable the reliable separation of proteins in the soluble and aggregated pool. However, were this unavailable, it may be necessary to use an abstract metric to quantify aggregation. Many abstract image metrics exist, including simple histogram-based metrics such as mean, variance, skewness and kurtosis, and Haralick Gray Level Colocalization Matrix (GLCM) texture descriptors [24,28]. Besides, a number of user-friendly machine learning programs have been developed, most notably CellProfiler and CellProfiler Analyst [29,30], that allow automatic identification of features of interest after initial training by the program user. As we will describe later, we demonstrate the validity of our extension of Ripley’s K-function as a theory-driven abstract index in quantifying protein aggregation using simulated and experimental ground-truth controls. In addition, we argue for the generalizability of our approach by performing a proof-of-concept analysis on a previously-published dataset, in order to validate its use in chromatin condensation quantification as well.

## Materials and Methods

### 1. Software used and data availability

All simulations and image processing codes were written and maintained in MATLAB (Version R2015a, The Mathworks Inc., USA). Preprocessing of protein aggregation images was done using ImageJ 1.49v and CellProfiler 2.1.1 [29,31]. The dataset and codes used for K-function calculation and validation are available in the supplementary materials (S1 and S2 Files).

### 2. Mathematical development

#### 2a. The original K-function

Ripley’s K is given by the equation:

$$K(r,n)=\frac{1}{\lambda }\cdot E\left(\text{number of events within radius }r\text{ of the }"\text{typical}"\text{ event}\right)$$ (1)

Where *E* is the expectation (i.e. “average”) number of events within radius *r*, *n* is the total number of events within the study area and *λ* is the intensity (density) of events, such that $\;\lambda \text{=}\frac{n}{\left|\Omega \right|}\;$ and |Ω| is the area of the study region (the entire field, including all potential event locations). Hence:

$$K(r,n)=\frac{\left|\Omega \right|}{n}\cdot \frac{1}{n-1}\cdot \sum_{i=1}^{n}\sum_{x\neq y}^{n}I_{r}(dxy)$$ (2)

Where *d*_*xy*_ is the distance between event locations *x* and *y* and *I*_*r*_ is an indicator function that has a value of 1 if the distance between locations *x* and *y* is less or equal to *r*. That is, $I_{r}(dxy)=\{\begin{array}{c} \text{1 if dxy}\leq r\\ \text{0 otherwise} \end{array}$

As with many image processing metrics, edge effects can be particularly challenging when it comes to Ripley’s K-function calculation. Given the definitions described earlier, consider the events that fall at a distance less than *r* from the edge of the image. Summing up the number of events within radius *r* of each of these “central” events would result in under-estimation; since the “empty” potential locations outside the study region may well have been populated with events were the field of view larger in size. Multiple edge correction methods have been devised for Ripley’s K-function; the most famous one was developed by Ripley himself, and is still used often in the literature. Ripley’s correction consists of dividing the number of events at a certain distance from the central event by the proportion of the circumference of a circle of the same radius that is included within the field of view. Since its original development, it was noted that this edge correction method is biased as it gives more weight to events that are farthest from the central event. One of the best and most widely accepted methods to correct for this bias was proposed by Besag [32]. Instead of correcting for events at each distance from the central event separately, Besag’s correction corrects for all events at once. That is, the overall number of events within a particular radius is divided by the proportion of the area of a circle of the same radius (centered on the central event) that is included within the field of view. For these reasons, we used Besag’s edge correction for all experiments in this paper.

Therefore,

$$K(r,n)=\frac{\left|\Omega \right|}{n(n-1)}\cdot \sum_{i=1}^{n}\frac{\pi r^{2}}{A_{xr}}\sum_{x\neq y}^{n}I_{r}(dxy)$$ (3)

A_*xr*_ is the area of the portion of circle *b*(*x*,*r*), centered on position *x* and having a radius *r*, that lies within the study region. Besag’s edge correction term is given by the equation:

$$A_{xr}=b(\text{x},r)\cap \left|\Omega \right|$$

Because Ripley’s K-function normalizes for the event density, its expectation at CSR is simply the area a circle of the same radius as that used to calculate the function. One problem with the original K-function is that it is not centered on zero, nor is it normalized to have a unit variance. While there are multiple versions of the K-function (including the L- and the H- functions [32,33]), we used the version proposed recently by Lagache *et al* [34], which also has a unit variance, and has the following equation:

$$\tilde{K}(r,n)=\frac{K(r,n)-\pi r^{2}}{\sqrt{\text{var}\left\{K(r,n)\right\}}}$$ (4)

Once the K-function of a particular field of view is obtained, it is compared to the upper and lower limits (typically, the 1^st^ and 99^th^ quantiles) of what its value would be at CSR. The theoretical determination of these “critical quantiles” proved to be especially difficult due to edge effects, and even though multiple attempts existed in the past, extensive Monte-Carlo simulations remained to be the only reliable method for critical quantile determination. It wasn’t until recently that Lagache and colleagues provided the first solid theoretical foundation for critical quantile determination, based on the Cornish-Fisher expansion [34]. As can be seen in Fig 1C, at CSR $\tilde{K}$ lies within the bounds of the theoretically-determined critical quantiles, while it lies outside these bounds when the events are clustered.

#### 2b. Extension to allow for event overlap

Our extension of Ripley’s K-function is as follows. We imagined each intensity unit to be one particle such that, in an 8-bit grayscale image, the saturation limit is 2^8^−1 = 255 particles per pixel. The number of particles around any particle within the same pixel = *P*_*x*_−1, where *P*_*x*_ is the intensity at pixel *x*. The number of particles around any particle in surrounding pixels within a circle of radius *r* = $\sum_{x\neq y}^{Np}I_{r}(dxy)$, where *Np* is the total number of pixels in the field of view and $I_{r}(dxy)=\{\begin{array}{c} P_{y}\text{ if }dxy\leq r\\ 0\text{ otherwise} \end{array}$.

Hence, the number of particles within radius *r* around each particle at pixel *x* =

$$P_{x}-1+\sum_{x\neq y}^{Np}I_{r}(dxy)$$ (5)

Summing up for all particles in a given pixel location *x* (and injecting Besag’s edge correction term), it follows that the total number of particles around particles at pixel *x* within radius *r* =

$$\frac{\pi r^{2}}{A_{xr}}\cdot P_{x}\left(P_{x}-1+\sum_{x\neq y}^{Np}I_{r}(dxy)\right)$$ (6)

Finally summing up over the whole field of view and normalizing to obtain K,

$$K(r,n)=\frac{\left|\Omega \right|}{n(n-1)}\cdot \sum_{i=1}^{Np}\frac{\pi r^{2}}{A_{xr}}\cdot P_{x}\left(P_{x}-1+\sum_{x\neq y}^{Np}I_{r}(dxy)\right)$$ (7)

Where *N*_*p*_ is the total number of pixels and *n* is the total intensity (i.e. total number of particles) over the whole field of view.

#### 2c. Different implementations of Besag’s edge correction term

**Fig 2:**
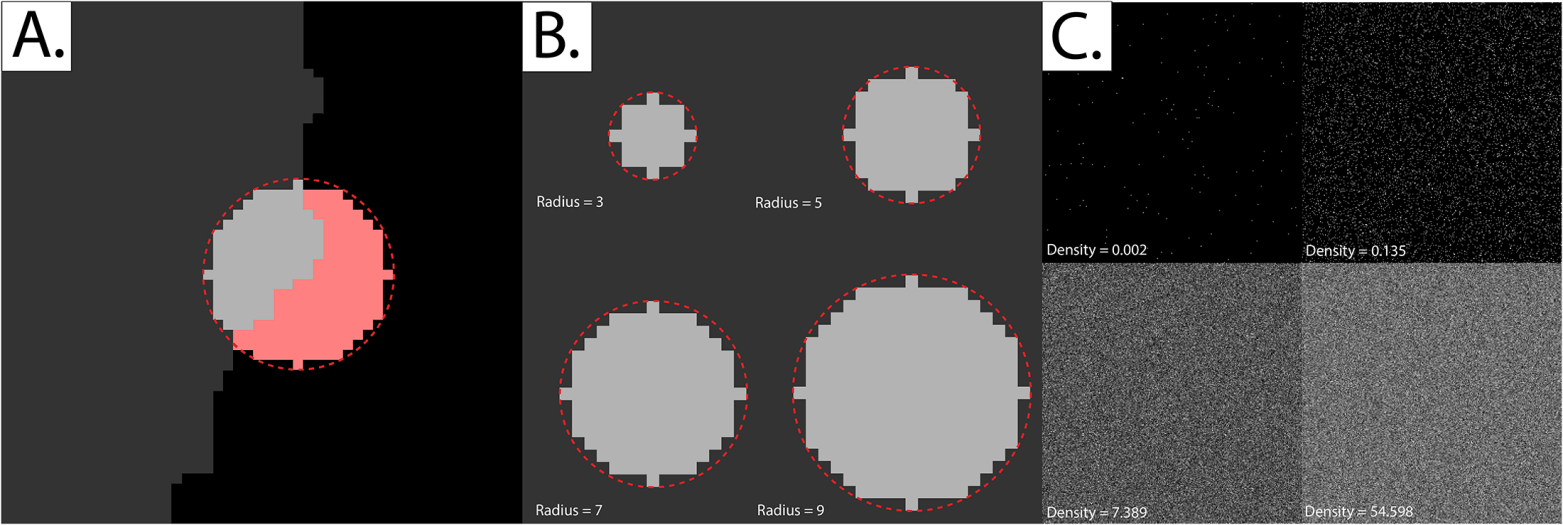
Approaching problematic edge effects at non-binary fields of view and small radii. *Panel A*: Three implementations of Besag’s edge correction term were tested. Note that the black region (black pixels) are considered to be outside the study region (i.e. the border between the grey and black area represents the edge of the study region), The implementations are as follows: i) Dividing the total number of pixels, located within a pre-defined radius from a given border pixel (light-gray area), by the total area of an ideal circle (area enclosed by the dotted red line). ii) Same as implementation i, but dividing by the total area of a non-ideal/pixelated circle (light-gray plus pink regions). iii) Same as implementation i, but applied for all pixels within the field of view. Hence, even for non-border pixels, the edge correction term would be obtained by dividing the area of the pixelated circle by the area of a perfect circle of the same radius. *Panel B*: The difference between an “ideal” circle and its pixelated counterpart at the four radii tested in this paper. *Panel C*: Complete Spatial Randomness (CSR) at a selected number of event densities (intensities). Each field of view measures 256 x 256 pixels. The image intensities were re-scaled for display purposes; the simulations used in Ripley’s K-function validation were set such that one intensity unit is akin to one event, at a saturation limit of 255 events per pixel (8-bit grayscale images).

To test the validity of our extension of Ripley’s K-function to grayscale (non-binary) fields of view, we devised the following validation workflow (illustrated in Fig 2).

We generated synthetic fields of view measuring 256 x 256 pixels and assigned particles at various densities to these pixels in a random manner, in order to create a homogenous Poisson distribution (CSR). The densities were exponentially increased and the K-function calculated at four different radii. In order to keep our method suitable for practical applications, particularly the quantitative ranking of mutant constructs in order of increasing protein aggregation, we used small-to-medium radii for K-function calculation. Two implementations of Besag’s edge correction term were tested, described by the following equations:

$$\text{Method I}:EdgeCorrection=\frac{\pi r^{2}}{b(\text{x},\text{r})\cap \left|\Omega \right|}$$

$$\text{Method II}:EdgeCorrection=\frac{b\left(x,r\right)}{b(\text{x},\text{r})\cap \left|\Omega \right|}$$

Both edge correction methods were applied to “border pixels”, where a border pixel is defined as any pixel located at a distance less than *r* from the margin of the field of view. Note that the area *b*(*x*,*r*) is actually less than *πr* ^2^ due to pixilation effects. As we will discuss in the results section, the two methods fail to maintain centralization of the K-function at high densities, and a simple adaptation where method I was applied to all pixels (not just border pixels) was needed.

### 3. Validation using simulations

#### 3a. Testing shape-dependence of the K-function

In order to adapt Ripley’s K-function for practical applications, zero pixels were ignored during edge correction, such that Regions of Interest (ROI’s) could be thresholded, with the background given a value of zero, before K-function calculation. Circular decimations, with a radius of 20 pixels, were randomly-scattered across a field of view with a minimum offset of one pixel. After the positions of decimations were determined, events were randomly distributed across all the other locations within the 256 x 256 pixel square field of view.

#### 3b. Testing correspondence between $\tilde{K}$ and the Aggregate-to-Diffuse Ratio (ADR)

We validated our approach by simulating the aggregation process and noting the change in $\tilde{K}$ as the ground-truth ADR values were gradually increased. Two modalities of increasing ADR were tested: (i) increasing the number of aggregates (clusters) while keeping the Signal-to-Background ratio (SBR) constant. (ii) increasing the SBR (effectively increasing the contrast) while keeping the number of aggregates constant.

Simulated 256 x 256 pixel fields of view were used in this experiment, with randomly-located circular clusters (aggregates) having a radius of 8 pixels. The signal was added to the background. In other words, pixels belonging to the aggregates had a value equal to the signal plus the background [35].

After particles were distributed across the field of view, the resulting image was convolved with a Gaussian blur filter having a diameter of 3 pixels and a sigma of 1 pixel, and Poisson noise was generated at high and low Signal-to-Noise ratios (SNR’s), simulating the Point Spread Function (PSF) and noise of a confocal microscope [36,37]. Poisson noise, being the main form of degradation occurring in confocal microscopy [35], was added by applying a Poisson noise generator to each pixel independently, with a lambda equal to the intensity of the pixel of interest [38]. $\tilde{K}$ was calculated for each image at a radius (r_k_) ranging between 2 and 15 pixels.

#### 3c. Characterizing aggregates

Kiskowski *et al* reported that a variable degree of correspondence existed between the radius at which Ripley’s K-function was maximized ($\left[{\tilde{K}}_{\text{max}}\right]$) and the true aggregate radius (r_agg_) [39]. They showed, using simulations of circular clusters of particles, that $\left[{\tilde{K}}_{\text{max}}\right]$ ranges between r_agg_ and 2r_agg_, depending on the separation distance between aggregates, as well as the presence of a diffuse (non-aggregated) pool of particles. We tested whether this still held true when particle overlap was allowed using the following method. Simulated 256 x 256 pixel fields of view were generated, with randomly-located circular clusters (aggregates) having a radius ranging between 2 and 10 pixels. $\tilde{K}$ was calculated for each image at a radius (r_k_) ranging between 1 and 35 pixels.

In addition, we illustrated the use of other, less speculative, techniques in characterizing aggregates. Two well-known methodologies may be used once the “abstract” measure of clustering (Ripley’s K-function) has been calculated: segmentation and granulometry. We generated 10 sets of 30 synthetic images, each containing four sets of 20 randomly-located aggregates having radii of 2, 4, 6 and 8 pixels. After particles were distributed across the field of view, the resulting image was convolved with a Gaussian blur filter and Poisson noise was added in the same manner described earlier.

Segmentation of the original images was achieved by passing the synthetic images through an imageJ pipeline consisting of the “Subtract Background” command [40], Auto-thresholding using the “MaxEntropy” method [41], followed by a “Close” operation with edge padding [42]. The granulometric profile was obtained by repeatedly opening the image using progressively larger radii and obtaining the pixel value sum of the resulting image [43,44].

We employed the bivariate similarity index described by Dima *et al* to illustrate the comparative accuracy and specificity of the two approaches described [45]. The bivariate similarity index, which is itself an extension of the widely used Jaccard Similary Index [46], relies on two measures: TET and TEE, given by the following equations:

$$\begin{array}{l} TET=\begin{array}{ccc} \frac{\left|T\cap E\right|}{\left|T\right|} & where & 0\leq TET\leq 1 \end{array}\\ TEE=\begin{array}{ccc} \frac{\left|T\cap E\right|}{\left|E\right|} & where & 0\leq TEE\leq 1 \end{array} \end{array}$$

Where T represents the number of non-zero pixels in the ground “Truth” mask, E represents the number of pixels in the “Estimate” mask, and *T*∩*E* is the number of pixels that are common to both the Truth and Estimate masks. Note that where there were no non-zero pixels in the Estimate mask, TEE was given a value of one [45].

### 4. Experimental validation

#### 4a. Quantifying protein aggregation

To further test the validity of our approach, we devised the following experimental setup. We created five constructs containing cells that have been co-transfected with two proteins, Ci and eGFP, which possess different intracellular localization behaviors. Ci (*Cubitus interruptus*) is the main transcription factor in the hedgehog signaling pathway. Ci generally exists in two forms in the cytoplasm: diffuse form and aggregated in large protein complexes with other signaling molecules such as Cos2, fused and su(fu) [23,47]. Complexed/aggregated Ci is readily sedimentable by ultracentrifugation [48], and has been shown to dimerize under certain conditions [49]. We found that forced constitutive dimerization of Ci, by replacing its first 440 amino acids with the dimerization domain of CGN4 (GCN4-Ci), results in a predominantly-punctate Ci distribution that appears mainly in the pellet fraction upon ultracentrifugation.

eGFP (Green Fluorescent Protein), on the other hand, is predominantly distributed in a diffuse manner when transfected into Clone 8 *Drosophila* cells. Hence, by controlling the co-transfection ratio and using the same tag and antibody for both GCN4-Ci and eGFP (in this case, HA- tag), we had a way to “artificially control” the ratio between aggregates and diffuse protein in a cell; an experimental “ground truth control” so-to-speak. Since the transfection efficiencies of Ci and eGFP are not equal, we confirmed the expression of both proteins by subcellular fractionation (differential centrifugation). Hence, the ratio between the pellet and soluble fractions represents the “true” ratio between aggregated and diffuse protein. We calculated the $\tilde{K}$ value for each cell by averaging out the $\tilde{K}$ values for different stacks obtained with confocal microscopy.

Co-transfection: Clone 8 cell lines [50] were obtained from (and maintained in accordance with) Indiana University's Drosophila Genomics Resource Center. The cells, which were passaged every three days, were cultured in T-25 flasks (Corning) using Complete M3 medium (Shields and Sang M3 insect medium Sigma S3652 with 2% FBS (Fetal Bovine Serum, Perbio Science), 100 units/ml penicillin G, 100 mg/ml streptomycin sulphate, 0.125 IU/ml insulin and 2.5% fly extract). Twenty-four hours before transfection, the cells were seeded onto 12-well plates at a density of 0.5x10^6^ cells per well. Afterwards, transfection with various amount of DNA was carried out using Effectene transfection reagent (QIAGEN) according to the manufacturer's protocol. All ROI's shown in this paper are from cells that express the following 3xHemagglutinin (HA) -tagged constructs: (i) GCN4-Ci and (ii) eGFP. The amount of DNA used for each of the five constructs is as follows:

Construct 1 –GCN4-Ci: 400ng, eGFP: 0ng;

Construct 2 –GCN4-Ci: 400ng, eGFP: 13.3ng;

Construct 3– GCN4-Ci: 400ng, eGFP: 33ng;

Construct 4 –GCN4-Ci: 330ng, eGFP: 67ng;

Construct 5 –GCN4-Ci: 0ng, eGFP: 300ng.

Confocal microscopy: Immunostaining was performed using mouse monoclonal anti-HA antibodies (1:500, 12CA5, Roche) followed by an anti-mouse secondary conjugated with Alexfluor594. Confocal microscopic images were acquired using LSM 510 (Zeiss Microscopy, Germany) and converted to 8-bit grayscale images for further processing.

Differential centrifugation: Cell were washed 3 times with ice-cold PBS and re-suspended in lysis buffer (10mM HEPES, ph7.4, 150mM NaCl, 0.5mM MgSO4, 1mM DTT and 1X Complete protease inhibitor (Roche)). Cells were lysed by passage through 27.5G needles 20 times. Unlysed cells and nuclei were removed by centrifugation at 800 g_avg_ for 5 mins. The pellet fraction was then obtained from centrifugation of the supernatant at 162,000 g_avg_ for 1 hr using a S140AT rotor (Hitachi), and the resulting supernatant was concentrated five times by methanol/chloroform precipitation (soluble fraction).

Image pre-processing: The raw images were convolved with a Gaussian blur operator with a sigma of 0.1 *μm* using ImageJ, in order to increase their SNR. Afterwards, all images were thresholded using Otsu’s method, which relies on minimizing the intra-class variance. Three-class Otsu thresholding was performed, with the middle class set to belong to the background [51]. The resultant images consisted of cytoplasmic protein (foreground) against a background of zeros.

$\tilde{K}$ values of stacks belonging to the same cell were “ensemble averaged” to get the “overall” $\tilde{K}$ for each cell. The “overall” $\tilde{K}$ results for all cells were then averaged to get the final $\tilde{K}$ metric for each construct. Error bars represent the standard error of the mean (SEM).

#### 4b. Quantifying chromatin condensation

We demonstrate the generalizability of the K-function by applying the algorithm to images of *Arabidopsis* plant cell nuclei that have been obtained by Poulet *et al* [52] and published as a dataset at: https://www.gred-clermont.fr/media/WorkDirectory.zip. Two nuclear phenotypes, originally described by Wang *et al* [53], were distinguished in the dataset: wild type (*wt*) and a double mutant lacking both *crwn1* and *crwn2* (shorthand for “crowded nuclei”) proteins, which are plant nucleoskeletal proteins. *Crwn1 crwn2* has been shown independently by Wang *et al* and Poulet *et al* to possess different phenotypic features, the most relevant of which is the smaller number of chromocentres than *wt* nuclei. Chromocentres are organized condensations of heterochromatin found in interphase nuclei. While Wang *et al* used manual methods to quantify the differences between *wt* and *crwn1 crwn2*, Poulet *et al* used semi-automated segmentation-based methods to do so. We calculated $\tilde{K}$ for each nucleus by calculating the $\tilde{K}$ value for each stack and averaging out the $\tilde{K}$ values to calculate the resultant $\tilde{K}$ value.

All images analyzed in this paper, whether simulated or experimental, were not subjected to intensity normalization, histogram equalization or any other contrast-altering process before $\tilde{K}$ calculation. All experimental images were 8-bit grayscale images, and all simulations were limited to the 8-bit dynamic range of 0–255 intensity units per pixel.

## Results and Discussion

### 1. Restoring the centralization of $\tilde{K}$

**Fig 3:**
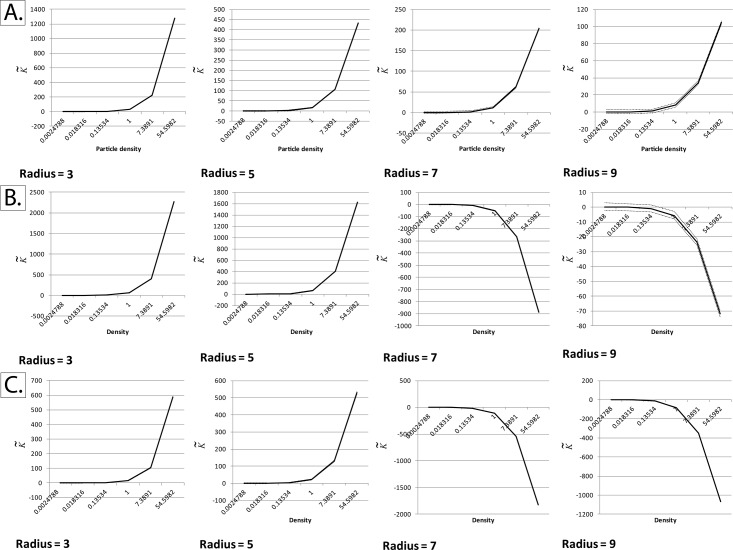
Unsuccessful implementations of Besag’s edge correction term at non-binary fields of view ($\tilde{K}$ with respect to event density at different radii). The normalized and centered K-function ($\tilde{K}$), as described by Lagache *et al*, was calculated at different particle intensities and radii using simulated square fields of view measuring 256 x 256 pixels, with a homogeneous Poisson distribution of particles (Complete Spatial Randomness). Densities are expressed in particles per pixel. The black line represents the mean $\tilde{K}$ and the dotted lines represent the upper and lower critical quantiles (Q01 and Q99). Each mean/quantile was determined using a set of 1000 simulations. *Panel A*: Applying Besag’s correction term only to edge pixels, by dividing the pixels within a given radius by the area of an ideal circle of the same radius. It can be seen that $\tilde{K}$ is no longer centered around zero at high particle intensities. *Panel B*: Same as panel A, but dividing by a pixelated circle rather than an ideal one. Like panel A, it can be seen that $\tilde{K}$ is no longer centered around zero at high intensities. *Panel C*: For comparison, $\tilde{K}$ was calculated without applying any edge correction terms. It can be seen that edge effects play a significant role at high particle intensities, making $\tilde{K}$ deviate remarkably from zero.

**Fig 4:**
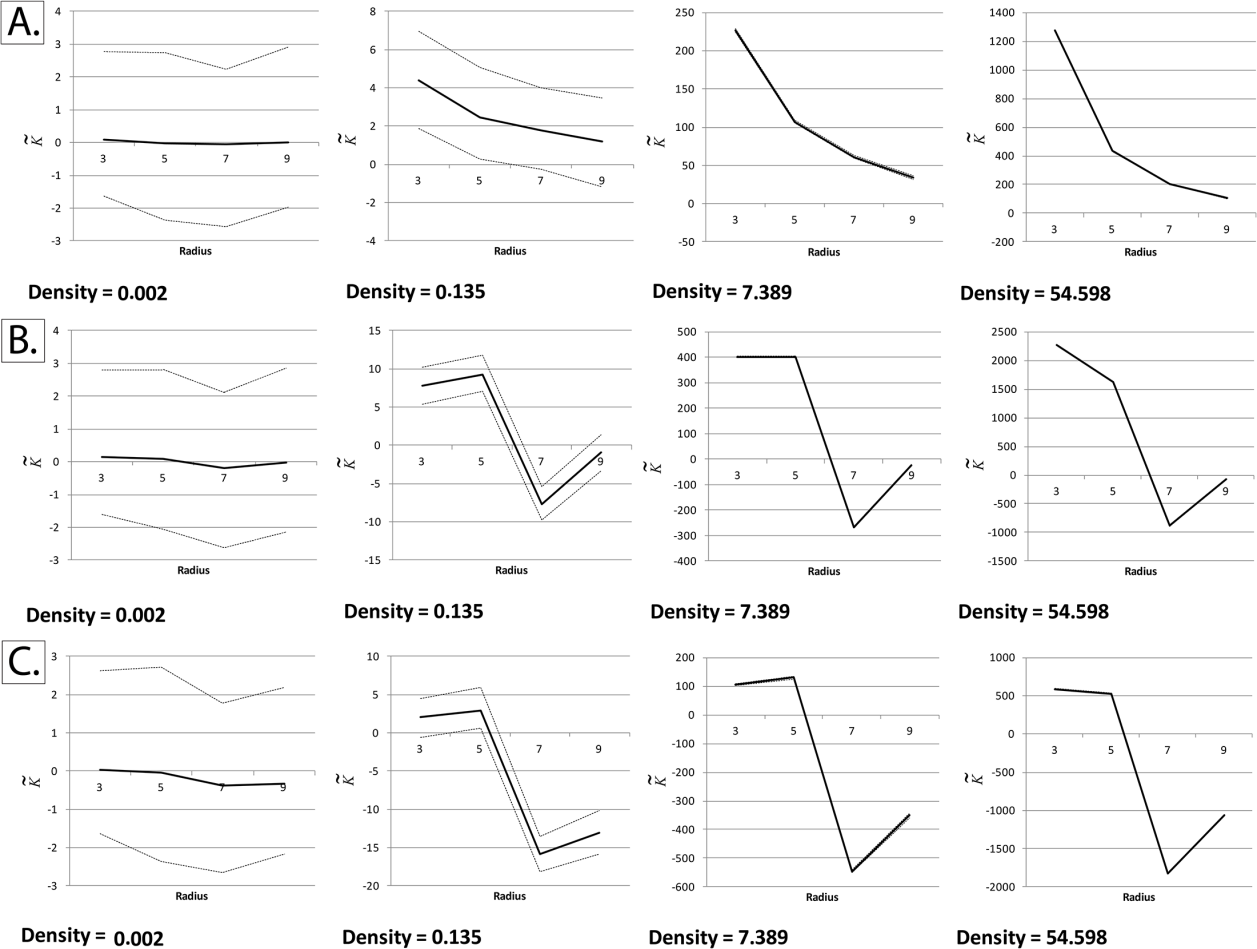
Unsuccessful implementations of Besag’s edge correction term at non-binary fields of view ($\tilde{K}$ with respect to radius at different event densities). The normalized and centered K-function ($\tilde{K}$), as described by Lagache *et al*, was calculated at different particle intensities and radii using simulated square fields of view measuring 256 x 256 pixels, with a homogeneous Poisson distribution of particles (Complete Spatial Randomness). Densities are expressed in particles per pixel. The black line represents the mean $\tilde{K}$ and the dotted lines represent the upper and lower critical quantiles (Q01 and Q99). Each mean/quantile was determined using a set of 1000 simulations. *Panel A*: Applying Besag’s correction term only to edge pixels, by dividing the pixels within a given radius by the area of an ideal circle of the same radius. It can be seen that $\tilde{K}$ is no longer centered around zero at high particle intensities. *Panel B*: Same as panel A, but dividing by a pixelated circle rather than an ideal one. Like panel A, it can be seen that $\tilde{K}$ is no longer centered on zero at high intensities. *Panel C*: For comparison, $\tilde{K}$ was calculated without applying any edge correction terms. It can be seen that edge effects play a significant role at high particle intensities, making $\tilde{K}$ deviate remarkably from zero.

**Fig 5:**
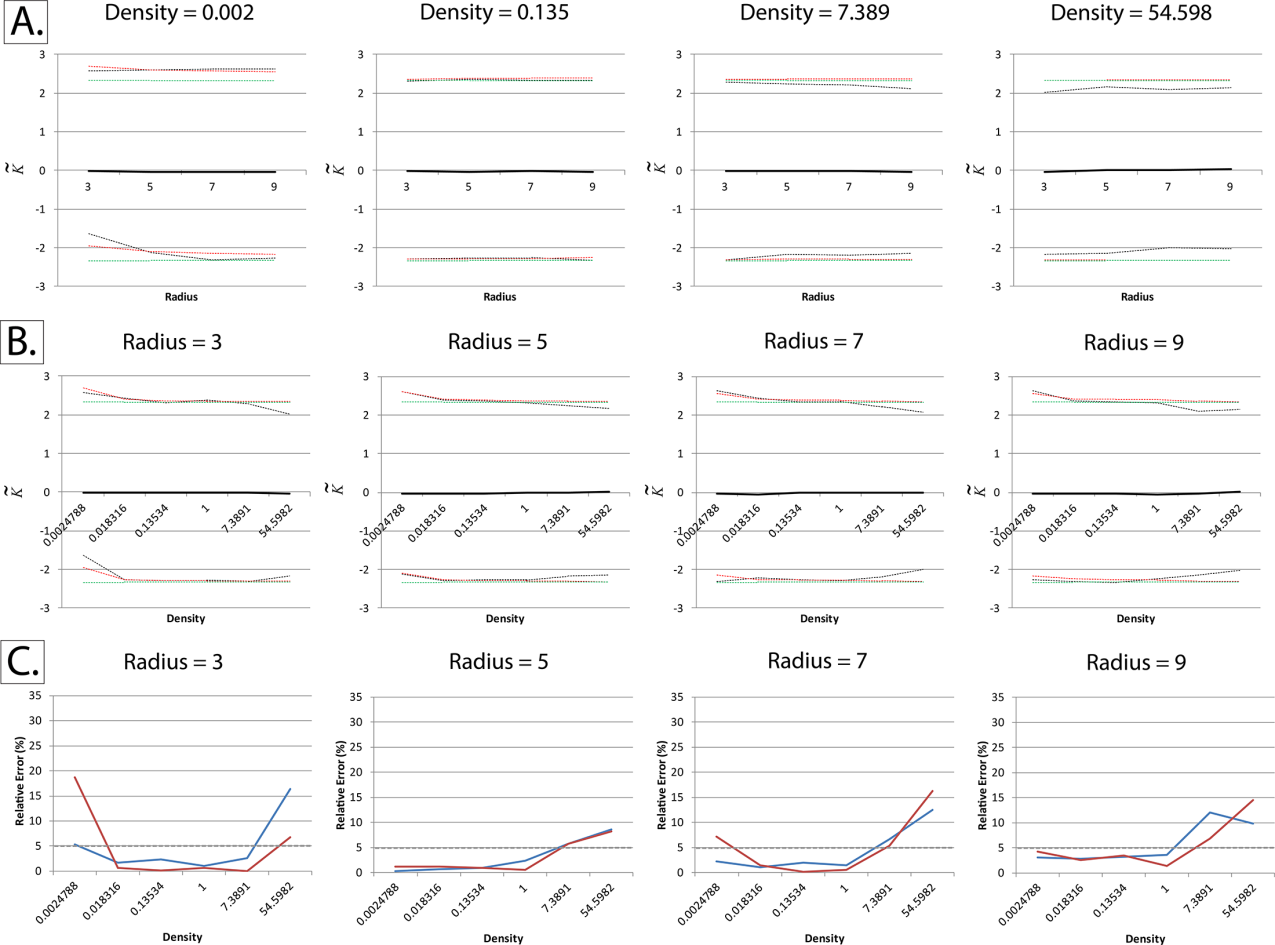
Successful adaptation of Besag’s boundary correction at non-binary fields of view. The normalized and centered K-function ($\tilde{K}$), as described by Lagache *et al*, was calculated at different particle intensities and radii using simulated square fields of view measuring 256 x 256 pixels, with a homogeneous Poisson distribution of particles (Complete Spatial Randomness). Densities are expressed in particles per pixel. Besag’s boundary correction was applied to all particles within the field of view and not just border pixels, as described in the text and in Fig 2. *Panels A and B*: The black line represents the mean $\tilde{K}$ and the dotted lines represent the upper and lower critical quantiles (Q01 and Q99). In order to ensure convergence, each mean/quantile was determined using a set of 25,000 simulations. The red dotted lines represent the Cornish-Fisher expansion used to estimate the critical quantiles, as validated by Lagache *et al*., while the green dotted lines represent the normal quantiles. Unlike the other unsuccessful implementations shown in Figs 3 and 4, mean $\tilde{K}$ remains centered on zero even at very high particle densities. *Panel C*: Quantifying relative error in the Cornish-Fisher expansion estimation of the empirically-determined critical quantiles.

We noticed that $\tilde{K}$ deviated markedly from zero when either one of the edge-correction methods described was utilized (Figs 3 and 4). We solved this issue by applying the edge correction term in method I not just to edge pixels, but to all pixels in the field of view (Fig 5). Essentially, what this means is that the “aggregation score” at every pixel is corrected for the difference between the “pixelated” circle forming the moving window and its “ideal” counterpart (Fig 2A and 2B).

The reason the former two methods failed while the latter worked is that at large particle densities, differences between the “pixelated” circle forming the moving window and its “ideal” counterpart become highly significant.

Consistent with Lagache *et al*. [34], the relative error is, for the most part, below the 5% benchmark. At very small radii (radius = 3) and very high densities (>7 particles per pixel), the relative error is higher than 5%, but still below 20%. At very high event densities, it can be seen that the empirical quantiles are closer to the mean than the Cornish-Fisher expansion, indicating that under these conditions the Cornish-Fisher expansion is a good “positive” (confirming clustering/dispersion), but a poorer “negative” (ruling out clustering/dispersion).

**Fig 6:**
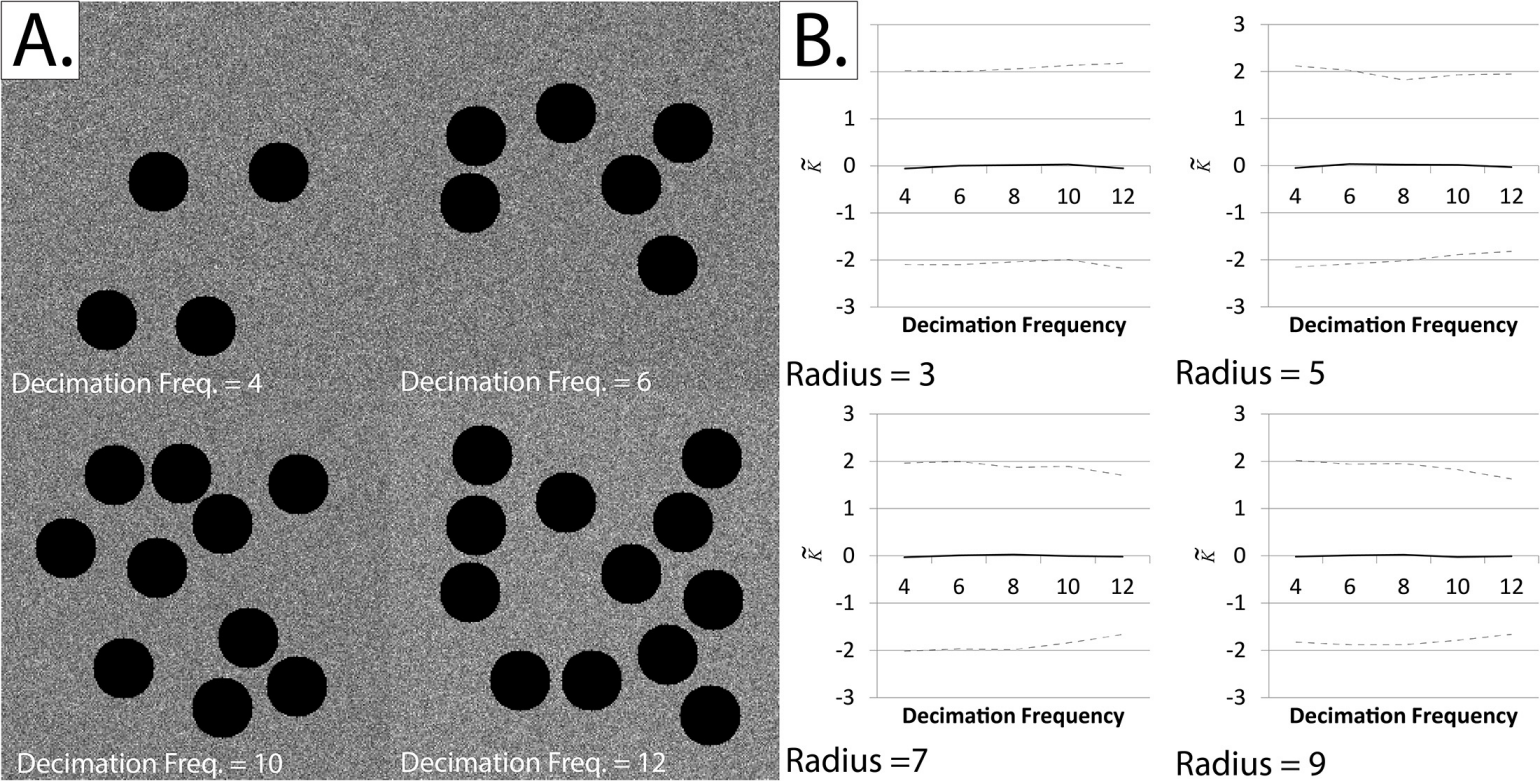
Testing the shape-dependence of our adaptation of Ripley’s K-function using repeated random decimation of simulated fields of view. Note that the black circular regions (black pixels) are considered to be outside the study region, and are generated to create artificial “edges”. *Panel A*: Sample simulated fields of view at decimation frequencies of 4, 6, 10 and 12. *Panel B*: The normalized and centered K-function ($\tilde{K}$) at various decimation frequencies. The black line represents the mean $\tilde{K}$ and the dotted lines represent the upper and lower critical quantiles (Q01 and Q99). Each mean/quantile was determined using a set of 1000 simulations. It can be seen that $\tilde{K}$ remains centered on zero regardless of the decimation frequency or radius at which $\tilde{K}$ was calculated.

Having shown that the extended form of Ripley’s K-function remains centralized at both small radii and high densities, we also showed that the modified form (which ignores zero pixels) is shape-invariant. Fig 6 shows that $\tilde{K}$ retains is centralization at zero even when a large proportion of the image has been decimated.

### 2. $\tilde{K}$ successfully ranks simulated images in the order of increasing ADR

**Fig 7:**
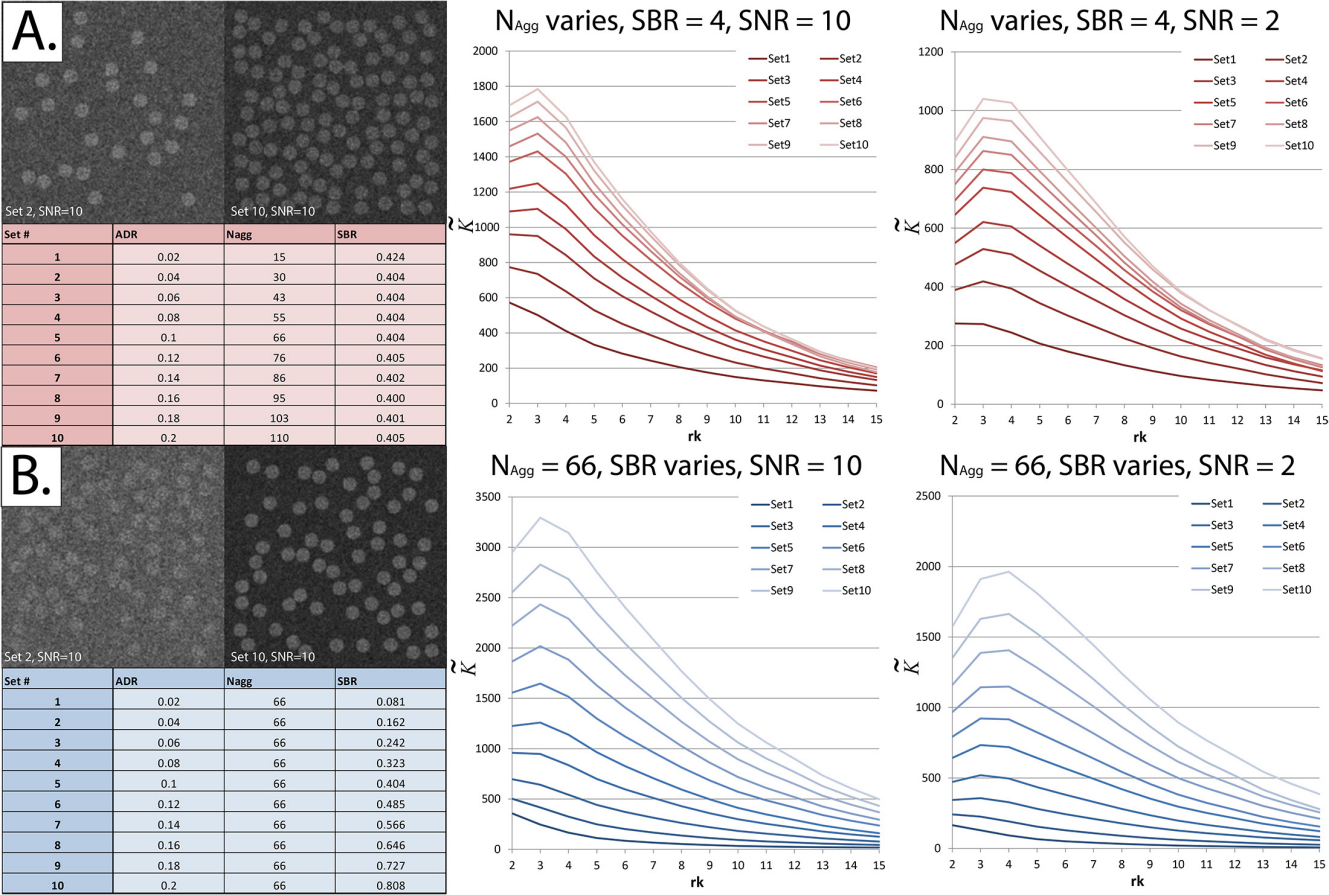
The normalized and centered K-function ($\tilde{K}$) increases as the Aggregate-to-Diffuse ratio (ADR) increases. Each $\tilde{K}$ result displayed represents the average of 30 simulated fields of view using the exact same experimental parameters. The *x*-axis in the right panels shows the radius at which $\tilde{K}$ was calculated (r_k_). *Panel A*: *Left*—ADR was varied (the proportion of particles allocated to aggregates) by varying the number of aggregates (N_agg_) while keeping the Signal-to-Background ratio (SBR) constant. Note that the signal is *added to* the background. Since the aggregates have a pre-defined radius, SBR was not the exact same, but varied subtly due to “quantization” effects as the number of aggregates is increased. *Right*—As ADR increases, $\tilde{K}$ increases. This remains true at SNR = 2. *Panel B*: *Left*—ADR was varied by varying the SBR while keeping N_agg_ constant. *Right*—As ADR increases, $\tilde{K}$ increases. This remains true at SNR = 2.

It can be seen in Fig 7 that $\tilde{K}$ increases when the proportion of particles assigned to aggregates (ADR) is increased, whether by increasing the number of aggregates or by increasing the number of particles assigned to each aggregate.

Our recommendation is to not consider $\tilde{K}$ to be a “hard” mathematical metric to quantify ADR itself. Instead, we prefer viewing $\tilde{K}$ as an abstract metric that increases as either component of clumping increases: spatial proximity of higher intensity pixels and intensity of aggregates relative to the background. Thus, $\tilde{K}$ is best used to rank images in the order of increasing aggregation under well-controlled conditions.

### 3. At very high densities, $\left[{\tilde{K}}_{\text{max}}\right]$ fails to correspond to the r_agg_

**Fig 8:**
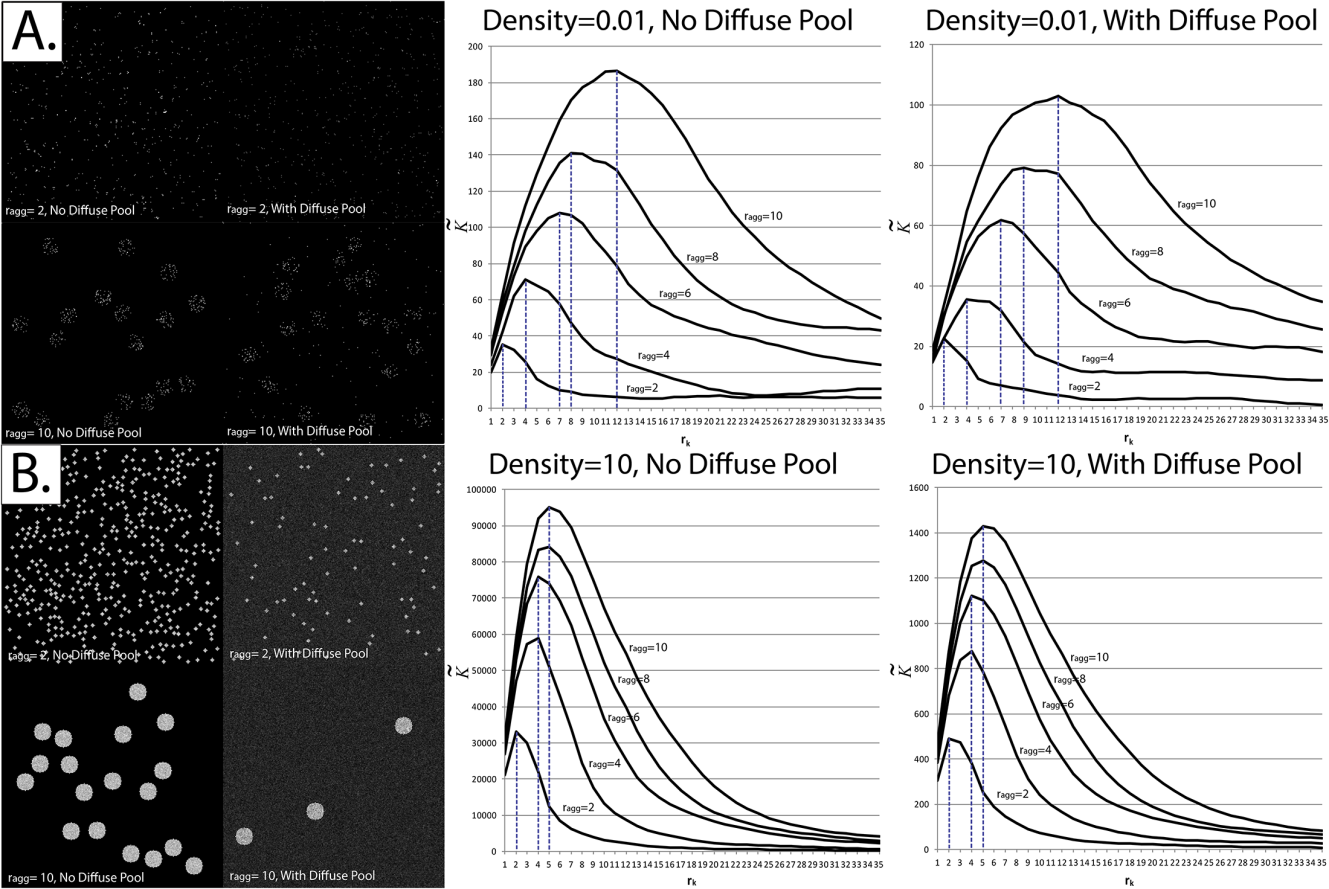
Correspondence between the radius at which $\tilde{K}$ is maximized and the radius of aggregates at binary and non-binary fields of view. Each $\tilde{K}$ result displayed represents the average of 30 simulated fields of view using the exact same experimental parameters. *Panel A*: Simulations were applied at very low event densities (0.01 particles per pixel) with and without a diffuse pool. *Left*—Sample simulated fields of view. The image intensities have been rescaled for display purposes. *Right*—Corresponding $\tilde{K}$ profile. *Panel B*: Simulations were applied at high event densities (10 particles per pixel) with and without a diffuse pool. For simulations where a diffuse pool is present, the Aggregate-to-Diffuse ratio (ADR) and Signal-to-Background ratio (SBR) were set at 0.05 and 3, respectively. *Left*—Sample simulated fields of view. *Right*—Corresponding $\tilde{K}$ profile.

Even though we were able to replicate the pattern reported by Kiskowski *et al* at low particle densities [39], the pattern broke down when the densities were increased to very large values. While it did remain true that as r_agg_ increased the radius at which $\tilde{K}$ was maximized (that is, $\left[{\tilde{K}}_{\text{max}}\right]$) also increased, $\left[{\tilde{K}}_{\text{max}}\right]$ no longer ranged between r_agg_ and 2r_agg_ at high particle densities (Fig 8).

Given the above results, the following question arises: are there any alternative methods that could be used to characterize the sizes of aggregates in grayscale images (other than the radius at which $\tilde{K}$ is maximized)? The answer is: yes, and in fact the existing methodologies for characterizing grayscale images are more abundant and intuitive than those of binary spatial distribution maps. Characterizing aggregates is arguably an easier process in grayscale images, since “concrete” statistical methods can be used, including segmentation and granulometry. For example, when traditional segmentation methods are applied to the image in Fig 8A, each particle is considered to be a separate “cluster”. On the other hand, at high particle densities (Fig 8B), a cluster would be successfully segmented.

**Fig 9:**
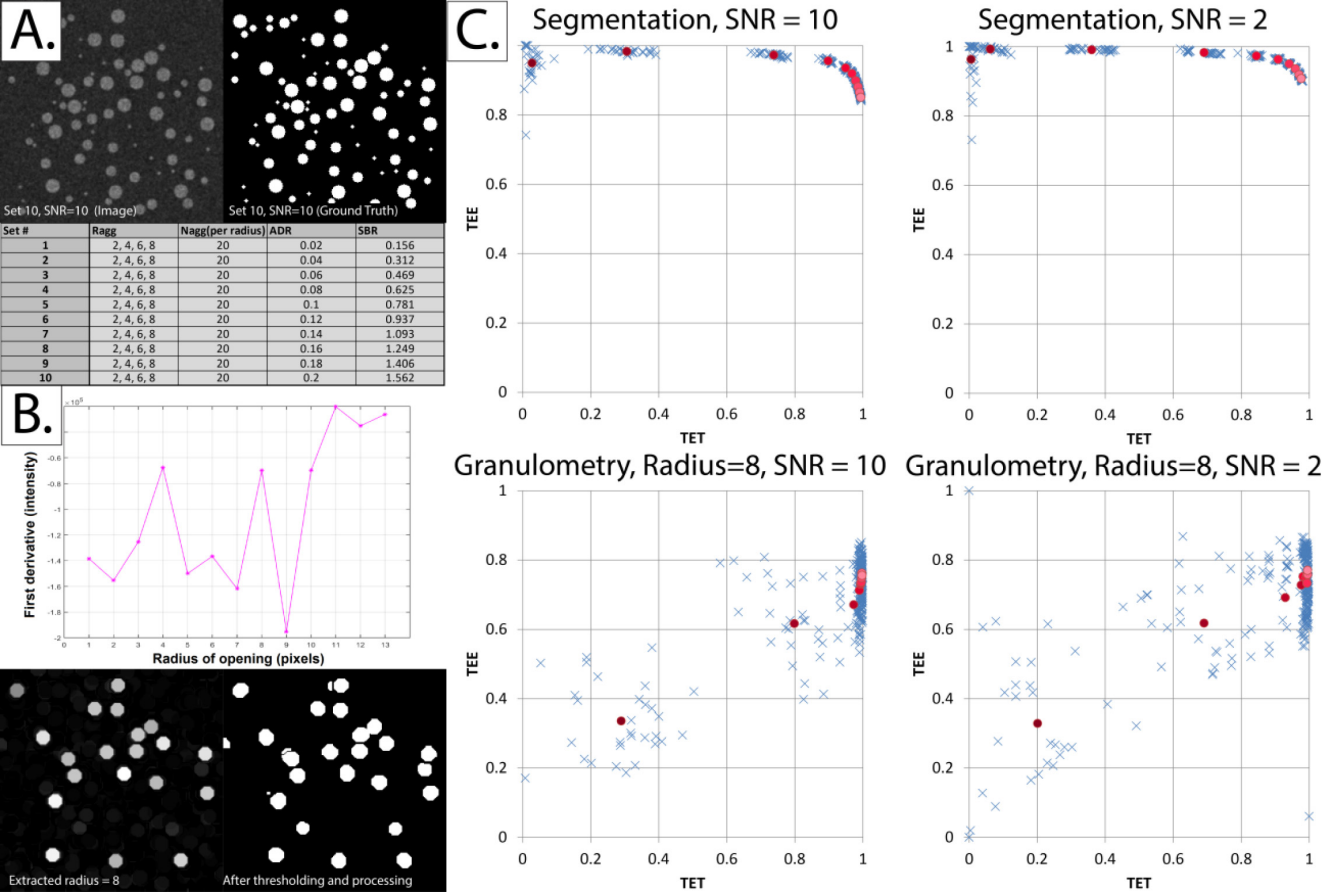
Alternative methodologies for characterizing aggregates at non-binary fields of view. *Panel A*: Sample simulated field of view, corresponding ground truth and parameters used in the 10 sets generated. *Panel B*: *Upper*—First-derivative of the granulometric profile of the sample field of view shown in Panel A. It can be seen that there are four major troughs at radii 2, 5, 7 and 9 approximately corresponding to the ground truth radii of the aggregates. *Lower*—Extracting aggregates with a radius of 8 pixels, by subtracting the opened image at a radius of 9 from that opened at a radius of 8. Further thresholding and processing may be performed (described in Panel C). *Panel C*: Demonstrating the effect of SBR on the accuracy and specificity of segmentation of aggregates. The blue crosses represent the TET and TEE values of individual simulated fields of view (see text for a description of how TET and TEE are calculated), while the means of Sets 1 through 10 are represented by different shades of red, from darker to lighter respectively. *Upper*—As SBR increases, TET increases. Notice the low TET values obtained for the Sets 1–3, especially at low SNR. *Lower*—The result of applying the same segmentation pipeline not to the original image but to aggregates of a single radius (8 pixels) obtained using granulometric analysis (as in Panel B). The TEE values are generally lower than those of the pipeline in the upper panel. However, this method offers better results at low SBR’s (Sets 1–3).

The comparative performance of segmentation and granulometry is illustrated in Fig 9. Generally-speaking, global thresholding has a more superior performance, but it performs poorer than granulometry at low SBR’s. Thus, even though $\left[{\tilde{K}}_{\text{max}}\right]$ cannot be used to approximate the size of clusters in grayscale images in the same manner that it could in binary spatial distributions, segmentation and granulometry are obvious alternatives. These methods offer the added benefit of spatial mapping of clusters as well as the ability to isolate clusters of variable sizes and shapes. The accuracy and specificity of such characterization process depends on the method used, as well as the image SBR and SNR values.

### 4. $\tilde{K}$ successfully ranks protein constructs in the order of increasing soluble (diffuse) fractions

**Fig 10:**
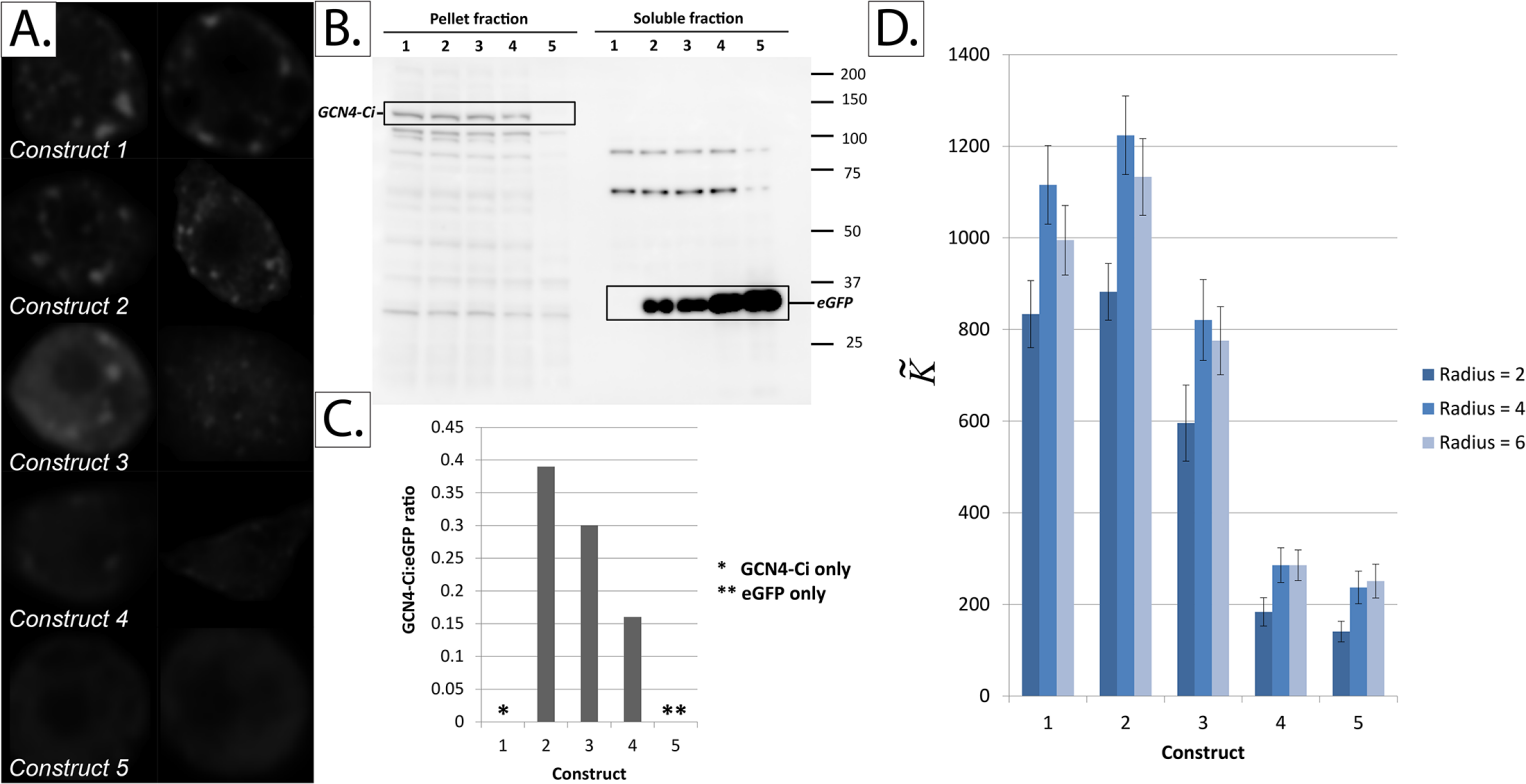
Quantifying protein aggregation in *Drosophila* Clone 8 cells co-transfected with GCN4-Ci (a predominantly-aggregated protein) and eGFP (a predominantly-soluble protein) in various ratios. *Panel A*: Sample images from the five constructs tested. *Panel B*: Western blot showing the results of subcellular fractionation of the five constructs. *Panel C*: Quantification of the western blot in panel B using ImageJ. *Panel D*: $\tilde{K}$ values for the five constructs tested. The number of cells analyzed in constructs 1, 2, 3, 4 and 5 is 17, 15, 16, 27 and 20 cells, respectively. Error bars represent the standard error of the mean (SEM).

It can be seen in Fig 10 that the value of $\tilde{K}$ successfully ranks four of the five constructs in the correct order of their GCN4-Ci:eGFP co-transfection ratios. Of course, this method has its limitations, and it can be seen that the subtle differences between the two most-aggregated constructs were not detected by $\tilde{K}$.

The results are consistent with our simulation results, particularly those in Fig 7A, as the main difference between various constructs is the increasing number (rather than intensity) of aggregates.

### 5. $\tilde{K}$ successfully ranks chromatin condensation in *wt* and *crwn1 crwn2 Arabidopsis* nuclei

**Fig 11:**
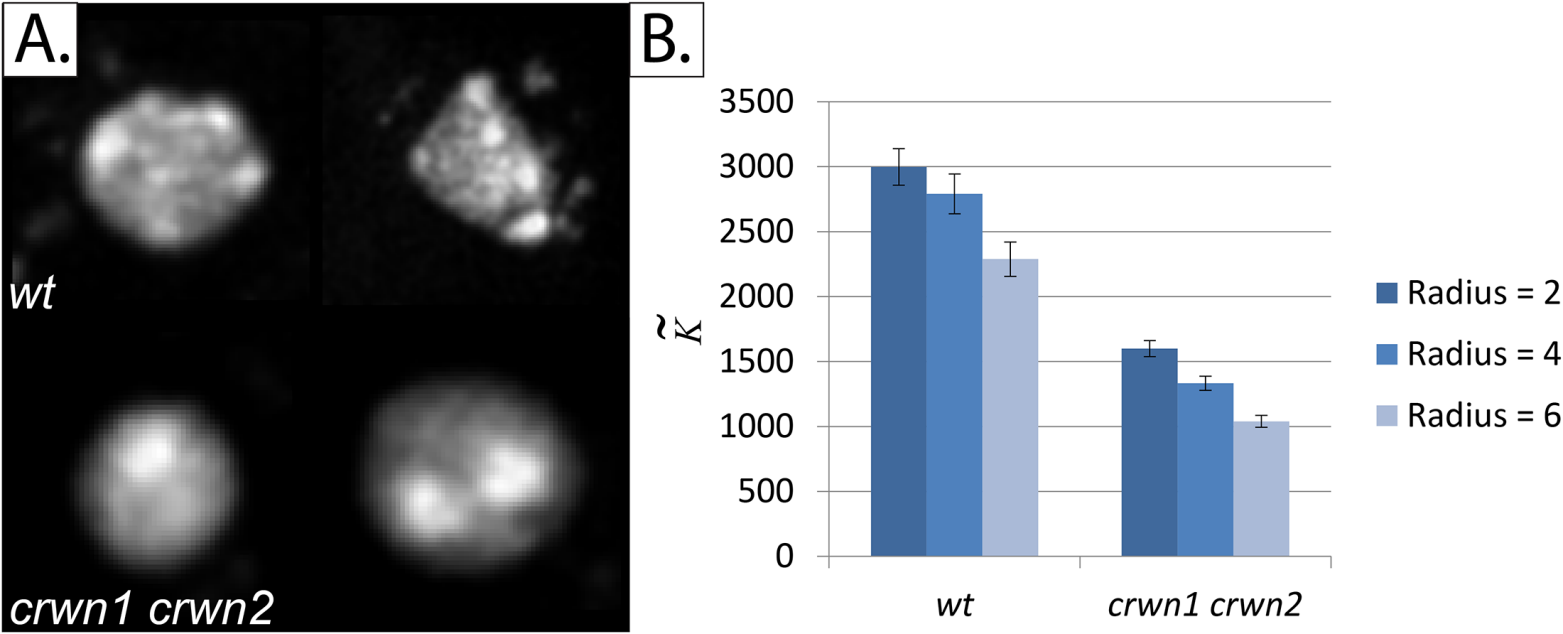
Quantifying chromatin condensation in *Arabidopsis* plant cells using the same dataset published by Poulet *et al* [52]. *Panel A*: Sample images from the two constructs tested: *wt* (wild type) and *crwn1 crwn2* mutant. *Crwn1 crwn2* mutant is known to have less chromocentres than *wt. Panel B*: $\tilde{K}$ values for the two constructs tested. The number of nuclei analyzed in the *wt* and *crwn1 crwn2* constructs is 38 and 39 nuclei, respectively. Error bars represent the standard error of the mean (SEM).

Our results, obtained using the same dataset as Poulet *et al*, confirm the findings of both Wang *et al* and Poulet *et al*; $\tilde{K}$ values of the *wt* construct were significantly higher than those of the *crwn1 crwn2* mutant (Fig 11). This can be attributed to the higher number of chromocentres in the *wt* construct (i.e. higher chromatin condensation state). One major advantage of using our approach is its applicability to large-scale batch processing of datasets. Manual methods, by comparison, are rather qualitative, often unreliable, and take a lot of time therefore limiting their utility in large-scale and real-time settings. Even semi-automated methods are time-consuming. Poulet *et al* reported that the time-limiting step in their analysis was the manual thresholding operation. Our method, which avoids segmentation altogether and uses an abstract index, avoids this step while still reliably reproducing the same results.

**Fig 12:**
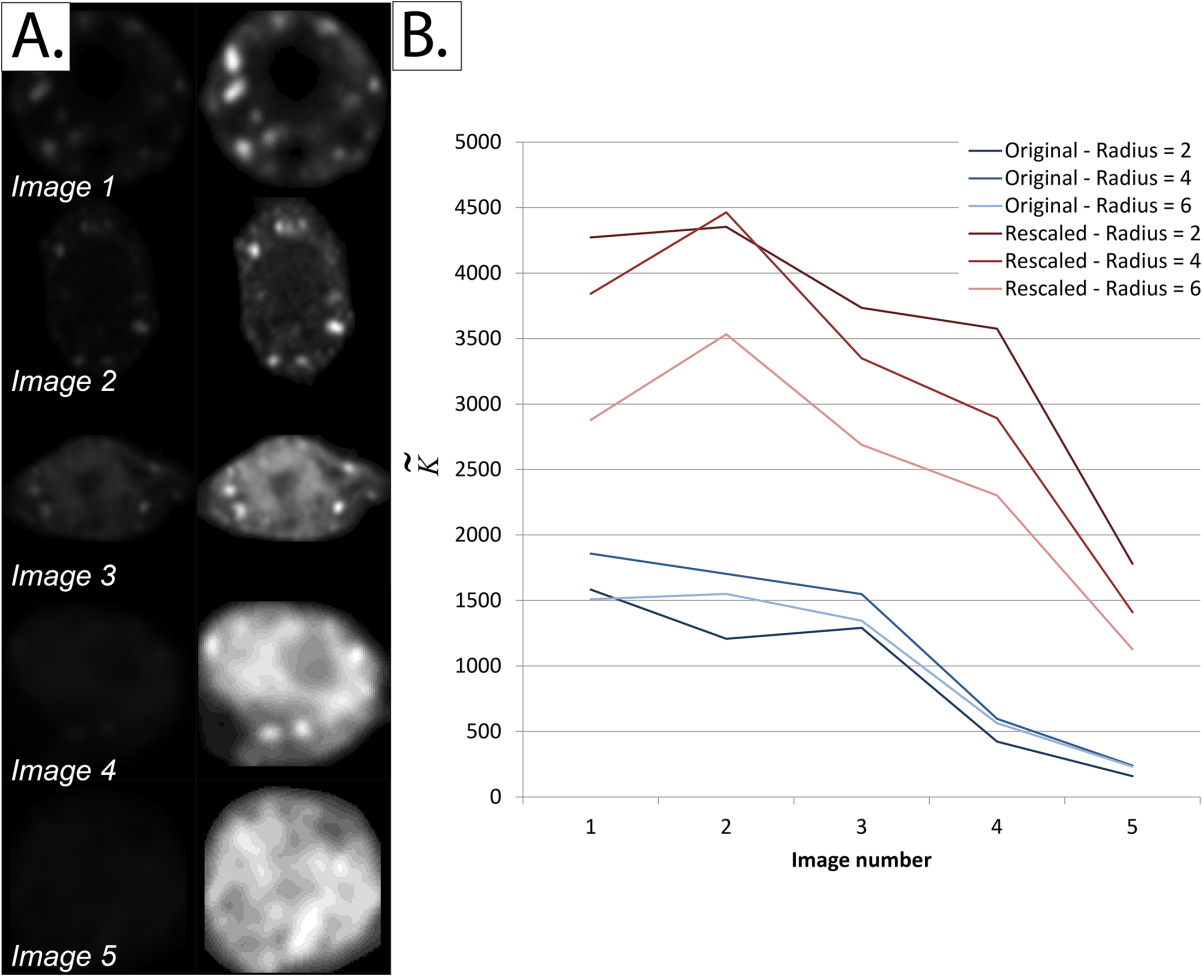
Distorting effect of intensity rescaling on $\tilde{K}$ values. *Panel A*: *Left*—Sample images from each of the five constructs described in Fig 10. *Right*—Same images after intensity normalization to occupy the full 8-bit dynamic range. *Panel B: $\tilde{K}$* values for the images in Panel A at three different radii (2, 4, and 6 pixels). Note the distorting effect of intensity normalization not only on the $\tilde{K}$ values, but also on the relative ranking of images.

Note that since $\tilde{K}$ is a spatially-variant metric that calculates the intensity distribution over an entire field of view, care should be taken when any type of intensity-altering process is applied, as this will almost certainly affect the resultant $\tilde{K}$ values (Fig 12). Another precaution to take is that images being compared should have the same bit-depth.

Moreover, it should be noted that, since $\tilde{K}$ is an abstract metric that quantifies clustering over an entire field of view, correct segmentation of the field of view against a background of zeros is quintessential to its success. For example, under-segmentation of the field of view may result in the whole cell being considered to be a “single big clump”, falsely increasing the $\tilde{K}$ value. By contrast, over-segmentation can result in falsely low $\tilde{K}$ values.

While the focus of this paper has been on protein and chromatin aggregation, it should be noted that the methods described could be used to quantify aggregation in any type of 2-D grayscale image without regard to the “material” being aggregated.

Not only does an abstract aggregation metric have implications in research, it could potentially be useful in diagnostic pathology as well. Indeed, computer-assisted diagnostics have gained a lot of attention over the past few years, and there are numerous efforts to integrate automated image processing and analysis methods into daily anatomic pathology practice. As a matter of fact, an image processing approach is probably the only realistic method to quantify aggregation in diagnostic pathology practice, which typically involves visual scanning of stained histopathological slides under brightfield microscopy.

## Conclusions

In conclusion, we extended the use of Ripley’s K-function to grayscale (non-binary) fields of view. A simple correction to Besag’s edge correction was presented, and was successful at restoring the function’s centralization at high densities. We showed that the extended form of the function is shape-invariant, and that previously-reported correspondence between the radius at which the K-function is maximized and true cluster radius break down at high densities. Simulations as well as co-transfection experiments were used to validate the function’s use as an abstract index of protein aggregation. In addition, proof-of-concept analysis was performed on a published chromatin condensation dataset to illustrate the generalizability of the extended form of the K-function.

## Supporting Information

S1 File Dataset used for the protein aggregation validation experiment. (RAR)

S2 File MATLAB functions and scripts used in this paper. (RAR)
